# Weight Gain and Body Composition Changes during the Transition of Thyroid Function in Patients with Graves' Disease Undergoing Radioiodine Treatment

**DOI:** 10.1155/2022/5263973

**Published:** 2022-07-18

**Authors:** Zhenqin Cai, Qiyu Chen, Yan Ling

**Affiliations:** Department of Endocrinology and Metabolism, Zhongshan Hospital, Fudan University, No.180 Fenglin Road, Shanghai 200032, China

## Abstract

**Objective:**

This study aimed at investigating the dynamic changes in the body weight and body composition in a group of patients with Graves' disease undergoing radioiodine therapy.

**Methods:**

Seventeen patients with Graves' disease undergoing ^131^I treatment and forty-three euthyroid controls were recruited. Body weight, BMI, and body composition via bioelectrical impedance were measured for the participants at baseline, hypothyroid stage, and euthyroid stage.

**Results:**

Body weight increased significantly during the transition from hyperthyroidism to euthyroidism. However, there were no significant changes in body fat %, lean mass %, and bone mineral %. The body weight of 9 patients at the euthyroid stage exceeded their premorbid weight, while the remaining 8 patients' weight did not exceed the premorbid weight. In the group with excessive weight gain, both body fat and lean mass increased significantly. However, in the group without excessive weight gain, only lean mass increased significantly. The patients with excessive weight gain had significantly higher body fat %, while lower lean mass % compared to patients without excessive weight gain at baseline and at the euthyroid stage. Moreover, body fat % of patients with excessive weight gain was significantly higher than that of controls, while lean mass % was significantly lower than that of controls. There was no difference of body fat % and lean mass % between patients without excessive weight gain and controls.

**Conclusion:**

^131^I treatment caused significant weight gain in patients with Graves' disease. An undesirable body composition at presentation may be a risk factor for excessive weight gain after hyperthyroidism treatment.

## 1. Background

It has long been accepted that thyroid hormone plays a crucial role in the control of energy homeostasis and can influence body mass and body composition significantly [1]. Hyperthyroidism is characterized by extensive weight loss due to the rapid loss of lean mass, while hypothyroidism always has weight gain, which is mainly caused by the retention of water in tissues [2]. Weight gain following the treatment of hyperthyroidism is usually considered a simple reaccumulation of premorbid weight. However, recent evidences suggest that there may be an excessive weight gain during the transition from hyperthyroidism to euthyroidism for many patients, which may unmask, or exacerbate, the predisposition towards obesity for some of them [3]. It has also been shown that the effects of different treatment modalities on weight gain following treatment for hyperthyroidism were not identical [4, 5]. One study compared the BMI of 1373 patients with overt hyperthyroidism from a large prospective secondary care cohort with the age- and sex-matched background population [6]. Both male and female patients had significant weight gain during the treatment of hyperthyroidism (the weight gain for men and women were 8.0 kg and 5.5 kg) and were at significantly increased risk of obesity compared to the background population [6]. Furthermore, radioiodine treatment was associated with more weight gain compared with antithyroid drug alone [6].

It has been reported that the overall mortality and mortality of cardiovascular disease increased significantly in patients diagnosed with hyperthyroidism [7–9]. Although at the moment, we are not very clear about the exact contribution of weight fluctuation observed in hyperthyroidism and its treatment to the excess mortality. Weight gain and development of obesity are thought to be one aspect of cardiovascular risk profile in this population. Besides characterizing weight changes, it is of interest to define the changes in the body composition when body weight increased significantly during the treatment of hyperthyroidism. It is important to make it clear whether there is deterioration of body composition during the process of weight gain. Several studies have analyzed the changes in the body composition after the treatment of hyperthyroidism, most of them used antithyroid drugs for treatment, but the results were inconsistent [10–15]. There is still controversy whether lean mass or fat mass or both are increased post-treatment of hyperthyroidism, and whether these changes occur concurrently or sequentially. One study prospectively observed the body weight changes of 75 patients treated for hyperthyroidism with radioiodine, and a subgroup of 9 patients had body composition measured by dual-energy X-ray densitometer (DEXA) [16]. They compared the body composition at baseline with that of 13 months post-treatment and concluded that weight gain occurred within the first year after radioiodine treatment is predominantly due to increased lean mass [16]. However, at the time of post-treatment body composition measurement, only 5 of the 9 patients were euthyroid, and the remaining 4 patients were hypothyroid [16].

The interindividual variability regarding the weight gain after hyperthyroidism treatment has been observed in many studies. Some patients had excessive weight gain compared with premorbid weight, while others only re-accumulated the disease-related weight loss and their post-treatment weight was not higher than their premorbid weight. It is not clear whether there are differences regarding body composition between patients with excessive weight gain and those without excessive weight gain, or where there is intrinsic relationship between body composition and weight gain in patients treated for hyperthyroidism.

Most hyperthyroid patients undergoing radioiodine therapy will experience a transition from hyperthyroidism to hypothyroidism and then become euthyroid with levothyroxine treatment, and thyroid function variation usually accompanies with prominent changes of body compartments. The objective of this study was to investigate the dynamic changes in the body weight and the body composition in a group of patients with Graves' disease undergoing radioiodine therapy. We also want to compare the differences of body composition between patients with post-treatment body weight exceeding the premorbid weight and those with post-treatment body weight not exceeding the premorbid weight.

## 2. Subjects and Methods

### 2.1. Study Design and Study Participants

Seventeen patients with Graves' disease undergoing radioiodine treatment were recruited from the Department of Endocrinology and Metabolism at Zhongshan Hospital affiliated to Fudan University. No patient had cardiac complications or other severe systemic diseases, or was taking medication likely to influence body weight or body composition, and fluid or electrolyte balance (e.g., corticosteroids and diuretics). They were evaluated before radioiodine therapy as the baseline. The dose of ^131^I administered was recorded. After radioiodine therapy, patients were followed up every 4–6 weeks. Supplementation with levothyroxine was immediately started when hypothyroidism developed (free T_4_ was below the lower limit of reference range). The patients were followed up until they became euthyroid and were on stable levothyroxine replacement for at least 3 months. At each clinic visit, patients were measured for body weight, waist circumference, hip circumference, body composition, and thyroid function when they were fast in the morning. The parameters obtained at baseline (hyperthyroid stage before ^131^I treatment), hypothyroid stage (the first time diagnosed as hypothyroidism after ^131^I treatment), and euthyroid stage (the last clinic visit when they became euthyroid and were on stable levothyroxine replacement for at least 3 months) were used in the analysis. To obtain the premorbid body weight, subjects were asked to recall their “usual” baseline weight, which have kept stable for several years before symptoms onset.

We also recruited euthyroid controls who attended the Outpatient Department of Internal Medicine of Zhongshan Hospital for health examination. The inclusion criteria were subjects with documented normal thyroid function in the recent 6 months. Subjects who had a history of abnormal thyroid function or a surgical history of thyroid gland, who used any medication that would affect thyroid function, body weight or body composition, and fluid or electrolyte balance (e.g., antithyroid drugs, thyroid hormone, amiodarone, corticosteroids, diuretics), who had severe systemic diseases, malignancy, or any acute intercurrent illness were excluded. Finally, forty-three euthyroid controls (11 men, 32 women) were included in the study. The study was approved by the Ethics Committee of Zhongshan Hospital, and all participants gave written informed consent.

### 2.2. Anthropometry and Body Composition

Weight was measured in light clothing on a calibrated electronic precision platform scale with the patient being fast in the morning. Height was measured with the patient barefoot using a precision meter. BMI was calculated as the body weight (kg) divided by the square of the body height (m^2^). Waist circumference was measured midway between the lower rib margin and the iliac crest in a standing position. The hip circumference was taken at the largest standing horizontal circumference of the buttocks. Body composition, including fat mass, lean mass, bone mineral content, visceral fat area, percentage of body fat (body fat %), percentage of lean mass (lean mass %), and percentage of bone mineral (bone mineral %), was measured in the fasting state via bioelectrical impedance using the body composition analyzer (InBody 720, Biospace China Inc).

### 2.3. Laboratory Measurements

Total thyroxine (TT_4_), total triiodothyronine (TT_3_), free thyroxine (fT_4_), free triiodothyronine (fT_3_), thyroid-stimulating hormone (TSH), thyroglobulin antibody (TGAb), thyroid peroxidase antibody (TPOAb), and thyrotropin receptor antibody (TRAb) were measured using the electrochemical luminescence method by the Modular E170 automatic electrochemiluminescence analyzer (Roche Diagnostics Ltd., Germany). The reference ranges were as follows: TT_4_ (66–181 nmol/L), TT_3_ (1.3–3.1 nmol/L), fT_4_ (12–22 pmol/L), fT_3_ (3.1–6.8 pmol/L), TSH (0.27–4.20 mIU/L), TGAb (<115 IU/mL), TPOAb (<34 IU/mL), and TRAb (<1.75 IU/L).

### 2.4. Statistical Analysis

Continuous variables were expressed as mean ± SD or median (interquartile range) and categorical variables as numbers. General linear model repeated measures or the Friedman test was used to compare differences of anthropometric and biochemistry parameters between different thyroid function states (baseline (hyperthyroidism), hypothyroidism, and euthyroidism). The group with body weight at the euthyroid state exceeding the premorbid weight was defined as the group with excessive weight gain, and the group with body weight at the euthyroid state not exceeding the premorbid weight was defined as the group without excessive weight gain. The independent-samples *t*-test or Mann–Whitney *U* test was used to compare differences of continuous variables between two groups. The chi-square test was used to compare differences of categorical variables between two groups. *P* < 0.05 was considered as significant. Analyses were performed using SPSS software version 26.0.

## 3. Results

### 3.1. Baseline Characteristics of the Patients

The baseline characteristics of the patients are presented in Table 1. We enrolled 17 patients with Graves' disease, with the mean age of 40.47 years. Among them, fifteen patients were women. The median duration of Graves' disease was 33 days. Fifteen patients had used antithyroid medications before radioiodine treatment. The mean body weight of them was 55.24 Kg, and the mean BMI was 21.27 Kg/m^2^. The mean premorbid weight was 58.59 Kg, and the mean premorbid BMI was 22.52 Kg/m^2^. One patient had hypertension, and none of them was diabetic. One patient was a smoker, and none of them had drinking habit. All patients had radioiodine treatment and were followed up until they became euthyroid and were on stable levothyroxine replacement for at least 3 months, and the mean follow-up duration was 446 days.

At the euthyroid state, nine patients' body weight exceeded their premorbid weight, while eight patients' body weight did not exceed their premorbid weight. We compared the baseline characteristics of the two groups. There was no significant difference between the two groups regarding age, gender, body weight, BMI, premorbid weight, premorbid BMI, waist circumference, duration of Graves' disease, proportion of antithyroid drug use, concentrations of thyroid hormones, thyroid autoantibodies, follow-up time, proportion of smokers, proportion of drinkers, proportion of hypertension, proportion of diabetes, radioiodine uptake rate, ^131^I dose, and duration from hyperthyroidism to euthyroidism.

### 3.2. Comparison of Anthropometric Parameters and Body Composition of the Patients at Different Thyroid Function States

Anthropometric parameters and body composition of the patients at different thyroid function states are presented in Table 2. In the whole population, the body weight was the lowest at the hyperthyroid state, increased significantly from hyperthyroidism to hypothyroidism, with no significant change from hypothyroidism to euthyroidism. The change in the BMI, waist circumference, and hip circumference was similar to the body weight. In terms of body composition, body fat, lean mass, and bone mineral content all increased significantly, with a significant increase from hyperthyroidism to hypothyroidism, but no significant change from hypothyroidism to euthyroidism. However, there was no significant change in body fat %, lean mass %, bone mineral %, and visceral fat area during the transition from hyperthyroidism to euthyroidism.

In the group with excessive weight gain, the body weight and BMI increased significantly from hyperthyroidism to hypothyroidism, and from hypothyroidism to euthyroidism. The changes in waist circumference and hip circumference were in a similar pattern as that of the body weight. Body fat, lean mass, and bone mineral content all increased significantly. However, body fat %, lean mass %, and bone mineral % did not change significantly. Although the visceral fat area increased, the change was not statistically significant.

In the group without excessive weight gain, the body weight and BMI increased significantly from hyperthyroidism to hypothyroidism, with no significant change from hypothyroidism to euthyroidism. There was a significant increase in the hip circumference, while no significant increase in the waist circumference. Although body fat increased, the trend of change was not significant. On the contrary, lean mass increased significantly. Body fat %, lean mass %, bone mineral %, and visceral fat area did not change significantly.

### 3.3. Comparison of Anthropometric Parameters and Body Composition between Patients with Excessive Weight Gain and Patients without Excessive Weight Gain

**Figure 1:**
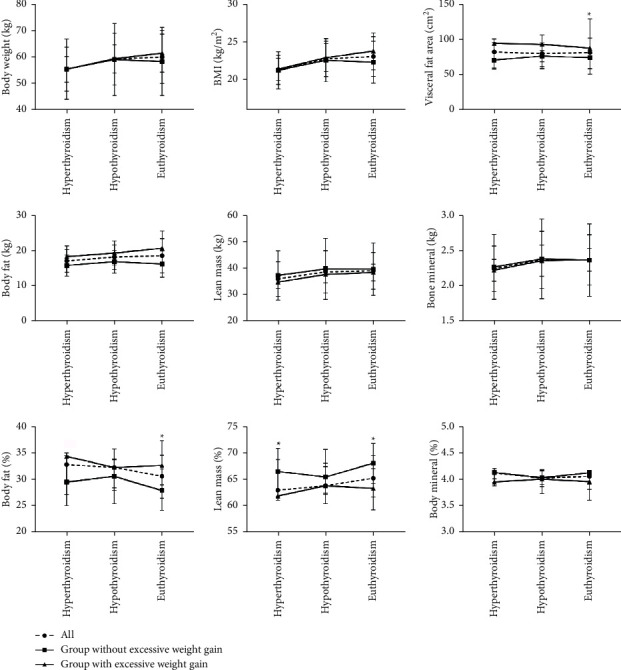
Comparison of body composition parameters between the patients with excessive weight gain and the patients without excessive weight gain under different thyroid function states. Body weight, BMI, visceral fat area, body fat, lean mass, bone mineral, and their percentages of body weight were compared between the patients with excessive weight gain and the patients without excessive weight gain under different thyroid function states. ^*∗*^ represents *p* < 0.05, indicating that there was a significant difference in the body composition parameter between the patients with excessive weight gain and the patients without excessive weight gain at a certain thyroid function state.

Comparison of anthropometric parameters and body composition between patients with excessive weight gain and patients without excessive weight gain is presented in Table 3 and Figure 1. At the hyperthyroid state, body weight, BMI, waist circumference, and waist-to-hip ratio were not significantly different between the two groups. However, body fat % in patients with excessive weight gain was significantly higher than those without excessive weight gain. On the contrary, lean mass % was significantly lower in patients with excessive weight gain.

At the hypothyroid state, there was no significant difference in the body weight, BMI, waist circumference, body fat %, and lean mass % between the two groups. The waist-to-hip ratio was significantly higher in patients with excessive weight gain than those without excessive weight gain.

At the euthyroid state, there was no significant difference in the body weight, BMI, waist circumference, and waist-to-hip ratio between the two groups. However, body fat % and visceral fat area in patients with excessive weight gain were significantly higher than those without excessive weight gain. On the contrary, lean mass % was significantly lower in patients with excessive weight gain.

### 3.4. Comparison of Anthropometric Parameters and Body Composition between Patients at Euthyroid State and Euthyroid Controls

Comparison of anthropometric parameters and body composition between patients at the euthyroid state and euthyroid controls is presented in Table 4. At the euthyroid state, there were no significant differences in age, gender, body weight, BMI, waist circumference, waist-to-hip ratio, bone mineral %, and visceral fat area between patients with excessive weight gain and control subjects. However, body fat % of patients with excessive weight gain was significantly higher than that of control subjects, while lean mass % of these patients was significantly lower than that of control subjects. Unlike patients with excessive weight gain, no significant difference was found between patients without excessive weight gain and control subjects.

## 4. Discussion

In this study, we prospectively observed the changes in the body weight and body composition in a group of patients with Graves' disease undergoing radioiodine treatment. During the transition from hyperthyroidism to euthyroidism, the body weight, BMI, waist circumference, and hip circumference of these patients increased significantly. The mean increase in the body weight and BMI was 4.61 ± 3.23 Kg and 1.75 ± 1.22 Kg/m^2^, respectively, when they achieved euthyroidism, and it translated into a 8.35% increase in their body weight. Both body fat and lean mass increased significantly, and both increments occurred concurrently and not in a sequential manner. However, body fat % decreased, while lean mass % increased during the follow-up period, although the differences between thyroid function states did not reach statistically significant. Therefore, there was no deterioration of body composition although significant body weight increase after radioiodine treatment in this group of patients with Graves' disease.

Many evidences have shown that weight gain following the treatment of hyperthyroidism is not a simple reaccumulation of premorbid weight. In a large cohort study by Torlinska et al., they recruited 1373 patients with overt hyperthyroidism and found that treatment for hyperthyroidism is associated with significant risks of obesity [6]. Compared to the background population, there was a 70% increased risk of obesity for male patients and a 30% increased risk for female patients [6]. In addition, they also found that treatment with ^131^I gained more weight than treatment with antithyroid drugs alone [6]. In our study, no patient was obese at baseline, and one patient became obese with a BMI over 28 Kg/m^2^ after ^131^I treatment. There is considerable interindividual variability regarding the weight gain after ^131^I treatment. The body weight of 9 patients at the euthyroid stage exceeded their premorbid weight, while the remaining 8 patients' weight did not exceed the premorbid weight. In the group with excessive weight gain, both body fat and lean mass increased significantly. However, in the group without excessive weight gain, only lean mass increased significantly. We found that body fat % and lean mass % were significantly different between the two groups both at baseline when they were hyperthyroid and at the euthyroid stage after the replacement of levothyroxine. The patients with excessive weight gain had significantly higher body fat %, while lower lean mass % compared to patients without excessive weight gain. Meanwhile, the visceral fat area of patients with excessive weight gain was also higher, which was borderline significant at baseline, and became significant at the euthyroid stage. We also compared the body composition of patients at the euthyroid state with that of euthyroid controls. We found that body fat % of patients with excessive weight gain was significantly higher than that of controls, while lean mass % was significantly lower than that of controls. We also found that there was no difference of body fat % and lean mass % between patients without excessive weight gain and controls. Our findings suggest that patients with excessive weight gain had worse body composition at baseline, which maintained until they became euthyroid. Although the premorbid weight and the weight at baseline were similar between the two groups of patients, the different body composition predisposed them to a different degree of weight gain. The transition from hyperthyroidism to euthyroidism may unmask the predisposition towards excessive weight gain in patients with undesirable body composition.

Previous studies have investigated the risk factors for excessive weight gain associated with hyperthyroidism treatments [4, 5, 17, 18]. Many potential risk factors have been recognized, including hyperthyroidism treatment modality, male gender, ethnicity, severity of thyrotoxicosis at presentation, development of iatrogenic hypothyroidism post-treatment, cause of hyperthyroidism, and pre-existing obesity [4, 5, 17, 18]. In our study, all patients were Chinese Han and were diagnosed with Graves' disease. There was no difference of gender, antithyroid drug use before ^131^I treatment, and severity of thyrotoxicosis at presentation between the two groups. All patients developed hypothyroidism after ^131^I treatment, and the duration from hyperthyroidism to euthyroidism was similar between the two groups. In addition, the total follow-up time was similar between the two groups. All patients had no pre-existing obesity. However, patients with excessive weight gain had higher body fat % but lower lean mass % than those without excessive weight gain at presentation, and these differences were maintained at the euthyroid stage in our study. This finding suggests that body composition at presentation may be a risk factor of excessive weight gain associated with ^131^I treatment of hyperthyroid patients. An undesirable body composition may predispose the subject to gain more weight and therefore may predict excessive weight gain after hyperthyroidism treatment. It is possible that hyperthyroidism and its reversal with subsequent treatment may unmask the predisposition towards excessive weight increase and accelerate this process at the same time.

The strengths of our study include the prospective study design, observation of weight, BMI, and body composition simultaneously, comparison with premorbid weight, and setting of control group. Nonetheless, our study has some limitations. First, the sample of our study is small. A study with larger sample should be carried out in the future to confirm our findings, and patients using different treatment modalities like antithyroid drug treatment and surgery should also be included in the future study. Besides, there was no overweight or obese patient at baseline in our study, making the results hard to be applied to these subjects. A study with overweight and obese patients should be carried out in the future. Second, subjective patient-reported premorbid weight may not be reliable. However, objective records of premorbid weight are usually unavailable, which will remain to be an important challenge in the future studies. Third, bioelectrical impedance analysis is less accurate than DEXA, which is considered the gold standard method for body composition assessment. However, DEXA is costly, time-consuming, and radiation exposed and is not suitable for repeated use. Lastly, the definition of the “excessive weight gain” of our study is too strict since weight varied with time. Our study is an exploratory pilot research, which needed further certification in larger samples. We will certainly improve the definition of “excessive weight gain” in the future study.

In conclusion, radioiodine treatment caused weight gain in patients with Graves' disease, and interindividual variability was observed on its impact on body weight when comparing post-treatment weight with premorbid weight. In patients with excessive weight gain, both at the presentation and at the post-treatment euthyroid stages, body fat % was higher, but lean mass % was lower compared with patients whose post-treatment weight does not exceed the premorbid weight. Our study suggests that an undesirable body composition may predispose the subject to gain more weight and therefore may predict excessive weight gain after hyperthyroidism treatment. Our findings provide a new risk factor of excessive weight gain following hyperthyroidism treatment, which should be confirmed in future studies with large samples.

## Figures and Tables

**Table 1:** Baseline characteristics of patients treated with ^131^I for hyperthyroidism.

	All	Group without excessive weight gain	Group with excessive weight gain	*P* value
Patient (n)	17	8	9	—
Female/male	15/2	6/2	9/0	0.21
Age (year)	40.50 ± 13.00	39.30 ± 11.60	41.60 ± 14.90	0.73
Premorbid weight (kg)	58.59 ± 10.59	59.88 ± 14.05	57.44 ± 6.97	0.67
Premorbid BMI (kg/m^2^)	22.52 ± 2.64	22.87 ± 2.88	22.20 ± 2.54	0.62
Body weight (kg)	55.24 ± 8.27	55.28 ± 11.39	55.21 ± 4.85	0.99
BMI (kg/m^2^)	21.27 ± 1.93	21.20 ± 2.45	21.30 ± 1.47	0.90
Waist circumference (cm)	76.55 ± 6.96	75.10 ± 8.17	77.83 ± 5.88	0.44
Hip circumference (cm)	92.66 ± 4.44	92.29 ± 6.25	92.99 ± 2.25	0.77
Waist-hip ratio	0.83 ± 0.06	0.81 ± 0.05	0.84 ± 0.07	0.40
Systolic blood pressure (mmHg)	132.40 ± 11.70	131.50 ± 8.20	133.00 ± 14.70	0.78
Diastolic blood pressure (mmHg)	74.90 ± 8.50	70.80 ± 6.50	78.60 ± 8.70	0.06
Smoking/nonsmoking (n)	1/16	1/7	0/9	0.47
Drinking/nondrinking (n)	0/17	0/8	0/9	—
Hypertension/nonhypertension (n)	1/16	0/8	1/8	1
Diabetes/nondiabetes (n)	0/17	0/8	0/9	—
Radioiodine uptake rate at 3 h (%)	61.20 ± 16.10	61.40 ± 11.70	61.10 ± 19.90	0.97
Radioiodine uptake rate at 24 h (%)	81.20 ± 9.50	81.00 ± 4.00	81.30 ± 12.90	0.94
Duration of disease (day)	33.00 (14.50–56.50)	37.50 (8.75–76.25)	30.00 (18.00–56.50)	0.89
Antithyroid drug use/no antithyroid drug use (n)	15/2	7/1	8/1	1
^131^I dose (mCi)	8.30 ± 1.00	8.30 ± 1.10	8.40 ± 1.10	0.88
T_3_ (nmol/L)	4.99 ± 1.80	4.52 ± 1.42	5.40 ± 2.07	0.33
T_4_ (nmol/L)	191.17 ± 34.95	184.00 ± 33.54	197.00 ± 36.90	0.44
fT_3_ (pmol/L)	21.29 ± 9.30	18.90 ± 7.31	23.41 ± 10.75	0.33
fT_4_ (pmol/L)	52.40 ± 16.30	48.60 ± 13.57	55.78 ± 18.52	0.38
TSH (uIU/ml)	<0.005	<0.005	<0.005	—
TGAb (IU/ml)	349.00 (34.70–463.50)	445.50 (61.00–502.50)	81.40 (25.70–443.00)	0.32
TPOAb (IU/ml)	167.20 (29.30–494.50)	261.50 (112.40–542.50)	91.00 (18.10–332.00)	0.24
TRAb (IU/L)	3.50 (2.60–15.70)	3.40 (2.30–14.90)	3.50 (3.10–14.50)	0.67
Duration from hyperthyroidism to euthyroidism (day)^a^	182.40 ± 81.00	189.40 ± 74.30	176.10 ± 90.60	0.56
Follow-up time (day)^b^	446.40 ± 72.80	419.80 ± 75.30	470.10 ± 65.60	0.16

^a^The time from admission for ^131^I treatment to the restoration of euthyroidism for the first time after levothyroxine replacement. ^b^The time from admission for ^131^I treatment to the end of the follow-up.

**Table 2:** Anthropometric parameters and body composition of patients treated with ^131^I for hyperthyroidism under different thyroid function states.

	All				Group without excessive weight gain				Group with excessive weight gain			
	Hyperthyroidism	Hypothyroidism	Euthyroidism	*P* value	Hyperthyroidism	Hypothyroidism	Euthyroidism	*P* value	Hyperthyroidism	Hypothyroidism	Euthyroidism	*P* value
fT_3_ (pmol/L)	21.29 ± 9.30	2.36 ± 0.68^a^	4.39 ± 0.68^ab^	<0.001	18.90 ± 7.31	2.29 ± 0.79^a^	4.25 ± 0.66^ab^	<0.001	23.41 ± 10.75	2.43 ± 0.60^a^	4.52 ± 0.71^ab^	<0.001
fT_4_ (pmol/L)	52.40 ± 16.30	5.79 ± 2.24^a^	18.41 ± 3.37 ^ab^	<0.001	48.60 ± 13.57	5.13 ± 2.30^a^	19.11 ± 4.17^ab^	<0.001	55.78 ± 18.52	6.39 ± 2.14^a^	17.78 ± 2.56^ab^	<0.001
TSH (uIU/ml)	<0.005	34.87 ± 31.98^a^	2.31 ± 2.58^ab^	0.001	<0.005	43.15 ± 37.75^a^	1.35 ± 1.20^ab^	0.02	<0.005	27.51 ± 25.86^a^	3.16 ± 3.21^ab^	0.02
Body weight (kg)	55.24 ± 8.28	59.07 ± 9.75^a^	59.85 ± 10.04^a^	<0.001	55.28 ± 11.39	58.91 ± 13.60^a^	58.16 ± 12.84^a^	0.007	55.21 ± 4.85	59.21 ± 5.30^a^	61.34 ± 7.19^ab^	<0.001
BMI (kg/m^2^)	21.27 ± 1.92	22.71 ± 2.31^a^	23.02 ± 2.61^a^	<0.001	21.20 ± 2.43	22.51 ± 2.87^a^	22.26 ± 2.75^a^	0.007	21.33 ± 1.48	22.88 ± 1.83^a^	23.70 ± 2.42^ab^	<0.001
Waist circumference (cm)	76.50 ± 6.96	78.68 ± 7.96^a^	80.06 ± 8.08^a^	0.01	75.10 ± 8.17	75.94 ± 10.10	77.19 ± 9.76	0.39	77.83 ± 5.88	81.11 ± 4.83^a^	82.61 ± 5.63^a^	0.03
Hip circumference (cm)	92.66 ± 4.44	95.32 ± 5.38^a^	95.71 ± 5.26^a^	<0.001	92.29 ± 6.25	95.56 ± 7.51^a^	93.75 ± 6.63	0.02	92.99 ± 2.25	95.11 ± 2.89^a^	97.44 ± 3.11^ab^	<0.001
Waist-hip ratio	0.83 ± 0.06	0.83 ± 0.06	0.84 ± 0.05	0.63	0.81 ± 0.05	0.79 ± 0.06	0.82 ± 0.05	0.20	0.84 ± 0.07	0.85 ± 0.04	0.85 ± 0.05	0.68
Body fat (kg)	17.10 ± 3.13	18.15 ± 3.42^a^	18.55 ± 4.74^a^	0.01	15.79 ± 2.92	16.86 ± 3.24^a^	16.20 ± 3.68	0.11	18.27 ± 2.98	19.29 ± 3.34^a^	20.63 ± 4.77^a^	0.004
Lean mass (kg)	35.90 ± 6.52	38.56 ± 8.01^a^	38.94 ± 6.92^a^	<0.001	37.23 ± 9.30	39.68 ± 11.54^a^	39.60 ± 9.86^a^	0.01	34.72 ± 2.45	37.57 ± 3.06^a^	38.34 ± 3.14^a^	<0.001
Bone mineral (kg)	2.24 ± 0.33	2.36 ± 0.41^a^	2.36 ± 0.36^a^	<0.001	2.26 ± 0.46	2.38 ± 0.57	2.36 ± 0.51	0.05	2.22 ± 0.16	2.35 ± 0.22^a^	2.37 ± 0.16^a^	<0.001
Body fat (%)	32.78(27.17–34.43)	32.24(27.96–33.81)	30.57(26.49–34.49)	0.94	29.49(25.12–33)	30.57(25.41–33.68)	27.89(24.19–31.95)	0.69	34.27(29.62–34.94)	32.24(28.37–35.72)	32.62(28.95–37.26)	0.64
Lean mass (%)	62.95(61.67–68.67)	63.78(62.15–67.96)	65.19(61.63–69.39)	0.59	66.46(62.87–70.79)	65.43(62.37–70.58)	67.99(64.14–71.72)	0.42	61.84(61.06–66.15)	63.78(60.43–67.38)	63.28(59.24–67.01)	0.64
Bone mineral (%)	4.12(3.95–4.15)	4.02(3.87–4.16)	4.06(3.81–4.16)	0.11	4.13(4.01–4.15)	4.03(3.91–4.16)	4.12(3.91–4.16)	0.20	3.95(3.86–4.12)	4.00(3.74–4.18)	3.96(3.61–4.08)	0.17
Visceral fat area (cm^2^)	80.08 ± 18.96	81.94 ± 20.61	83.80 ± 27.93	0.42	71.61 ± 14.52	74.55 ± 17.22	69.81 ± 18.43	0.28	87.61 ± 19.98	88.51 ± 22.07	96.23 ± 29.89	0.15

^a^Compared with hyperthyroidism, *P* < 0.05; ^b^Compared with hypothyroidism, *P* < 0.05.

**Table 3:** Comparison of anthropometric and body composition parameters between the group with excessive weight gain and the group without excessive weight gain under different thyroid function states.

	Hyperthyroidism			Hypothyroidism			Euthyroidism		
	Group without excessive weight gain	Group with excessive weight gain	*P* value	Group without excessive weight gain	Group with excessive weight gain	*P* value	Group without excessive weight gain	Group with excessive weight gain	*P* value
fT_3_ (pmol/L)	18.90 ± 7.31	23.41 ± 10.75	0.33	2.29 ± 0.79	2.43 ± 0.60	0.67	4.25 ± 0.66	4.52 ± 0.71	0.43
fT_4_ (pmol/L)	48.60 ± 13.57	55.78 ± 18.52	0.38	5.13 ± 2.30	6.39 ± 2.14	0.26	19.11 ± 4.17	17.78 ± 2.56	0.43
TSH (uIU/ml)	<0.005	<0.005	1	43.15 ± 37.75	27.51 ± 25.86	0.33	1.35 ± 1.20	3.16 ± 3.21	0.15
Body weight (kg)	55.28 ± 11.39	55.21 ± 4.85	0.99	58.91 ± 13.60	59.21 ± 5.30	0.96	58.16 ± 12.84	61.34 ± 7.19	0.55
BMI (kg/m^2^)	21.20 ± 2.43	21.33 ± 1.48	0.90	22.51 ± 2.87	22.88 ± 1.83	0.76	22.26 ± 2.75	23.70 ± 2.42	0.27
Waist circumference (cm)	75.10 ± 8.17	77.83 ± 5.88	0.44	75.94 ± 10.10	81.11 ± 4.83	0.22	77.19 ± 9.76	82.61 ± 5.63	0.20
Hip circumference (cm)	92.29 ± 6.25	92.99 ± 2.25	0.77	95.56 ± 7.51	95.11 ± 2.89	0.88	93.75 ± 6.63	97.44 ± 3.11	0.18
Waist-hip ratio	0.81 ± 0.05	0.84 ± 0.07	0.40	0.79 ± 0.06	0.85 ± 0.04	0.03	0.82 ± 0.05	0.85 ± 0.05	0.29
Body fat (kg)	15.79 ± 2.92	18.27 ± 2.98	0.11	16.86 ± 3.24	19.29 ± 3.34	0.15	16.20 ± 3.68	20.63 ± 4.77	0.05
Lean mass (kg)	37.23 ± 9.30	34.72 ± 2.45	0.48	39.68 ± 11.54	37.57 ± 3.06	0.54	39.60 ± 9.86	38.34 ± 3.14	0.74
Bone mineral (kg)	2.26 ± 0.46	2.22 ± 0.16	0.79	2.38 ± 0.57	2.35 ± 0.22	0.90	2.36 ± 0.51	2.37 ± 0.16	0.98
Body fat (%)	29.49 (25.12–33)	34.27 (29.62–34.94)	0.02	30.57 (25.41–33.68)	32.24 (28.37–35.72)	0.25	27.89 (24.19–31.95)	32.62 (28.95–37.26)	0.04
Lean mass (%)	66.46 (62.87–70.79)	61.84 (61.06–66.15)	0.03	65.43 (62.37–70.58)	63.78 (60.43–67.38)	0.25	67.99 (64.14–71.72)	63.28 (59.24–67.01)	0.04
Bone mineral (%)	4.13 (4.01–4.15)	3.95 (3.86–4.12)	0.23	4.03 (3.91–4.16)	4.00 (3.74–4.18)	0.70	4.12 (3.91–4.16)	3.96 (3.61–4.08)	0.11
Visceral fat area(cm^2^)	71.61 ± 14.52	87.61 ± 19.98	0.08	74.55 ± 17.22	88.51 ± 22.07	0.17	69.81 ± 18.43	96.23 ± 29.89	0.05

**Table 4:** Comparison of anthropometric and body composition parameters between patients at the euthyroid state and euthyroid controls.

	Group without excessive weight gain	Group with excessive weight gain	Control group
Female/Male (n)	6/2	9/0	32/11
Age (year)	39.3 ± 11.6	41.6 ± 14.9	45.5 ± 14.6
Body weight (kg)	58.16 ± 12.84	61.34 ± 7.19	62.94 ± 12.52
BMI (kg/m^2^)	22.26 ± 2.75	23.70 ± 2.42	22.63 ± 3.05
Waist circumference (cm)	77.19 ± 9.76	82.61 ± 5.63	82.71 ± 10.57
Hip circumference (cm)	93.75 ± 6.63	97.44 ± 3.11	97.97 ± 6.41
Waist–hip ratio	0.82 ± 0.05	0.85 ± 0.05	0.84 ± 0.07
Body fat (kg)	16.20 ± 3.68	20.63 ± 4.77	18.13 ± 5.29
Lean mass (kg)	39.60 ± 9.86	38.34 ± 3.14^a^	42.26 ± 9.60
Bone mineral (kg)	2.36 ± 0.51	2.37 ± 0.16	2.59 ± 0.53
Body fat (%)	27.89 (24.19–31.95)	32.62 (28.95–37.26)^a^	28.65 (24.08–34.65)
Lean mass (%)	67.99 (64.14–71.72)	63.28 (59.24–67.01)^a^	67.31 (61.39–72.80)
Bone mineral (%)	4.12 (3.91–4.16)	3.96 (3.61–4.08)	4.06 (3.90–4.37)
Visceral fat area (cm^2^)	69.81 ± 18.43	96.23 ± 29.89	84.34 ± 29.73

^a^ Compared with the control group, *P* < 0.05.

## Data Availability

The data used to support the findings of this study are available from the corresponding author upon request.
